# Supplementary material for: Sequence of abrupt transitions in Antarctic drainage basins before and during the Mid-Pleistocene Transition
Source: Nat Commun. 2025 Nov 24;16:10391. doi: 10.1038/s41467-025-65375-x (PMC12644747; doi:10.1038/s41467-025-65375-x)
Supplement: Supplementary file 1 — Supplementary Information [file 41467_2025_65375_MOESM1_ESM.pdf]

Supplementary information: Sequence of abrupt transitions in Antarctic drainage basins before and during the Mid-Pleistocene Transition

Christian Wirths*[1,2], Antoine Hermant[1,2], Christian Stepanek[3], Thomas F. Stocker[1,2], and Johannes C. R. Sutter[1,2]

[1]Climate and Environmental Physics, University of Bern, Bern, Switzerland
[2]Oeschger Centre for Climate Change Research, University of Bern, Bern, Switzerland
[3]Alfred Wegener Institute, Helmholtz Center for Polar and Marine Research, Bremerhaven, Germany

1 Extended Figures

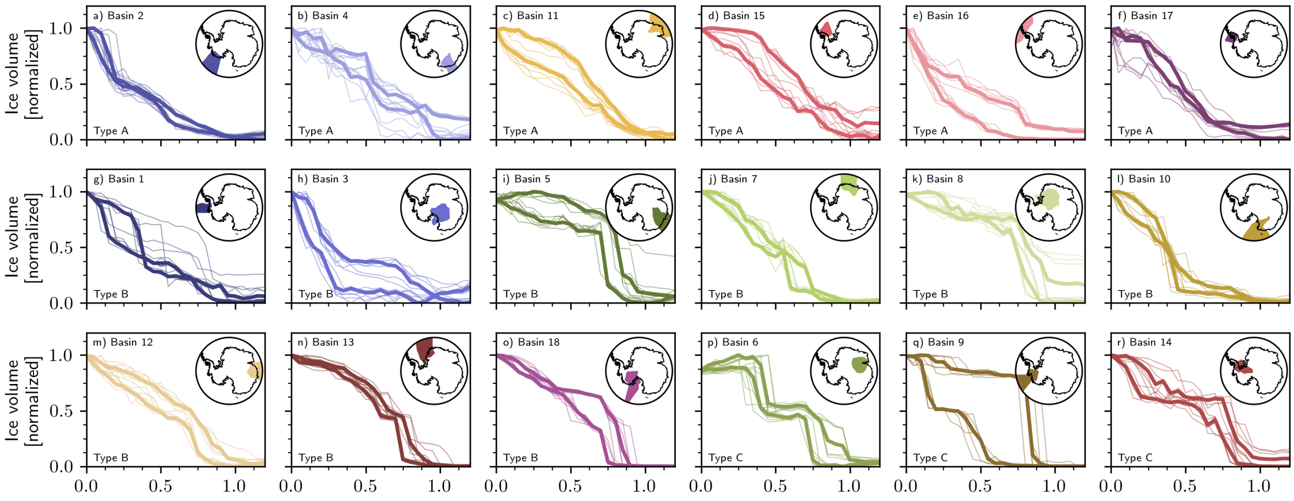

Figure S1: Hysteresis of grounded ice volume for all Antarctic basins for climate conditions between the Pliocene and Last Glacial Maximum. Thick lines indicate median and thin lines show individual parameter suite grounded ice volume. Ice volume is normalized for every basin individually to fit a common y-axis. Ice volume values for the individual basins have been normalized using min-max scaling.

*Corresponding author: christian.wirths@unibe.ch

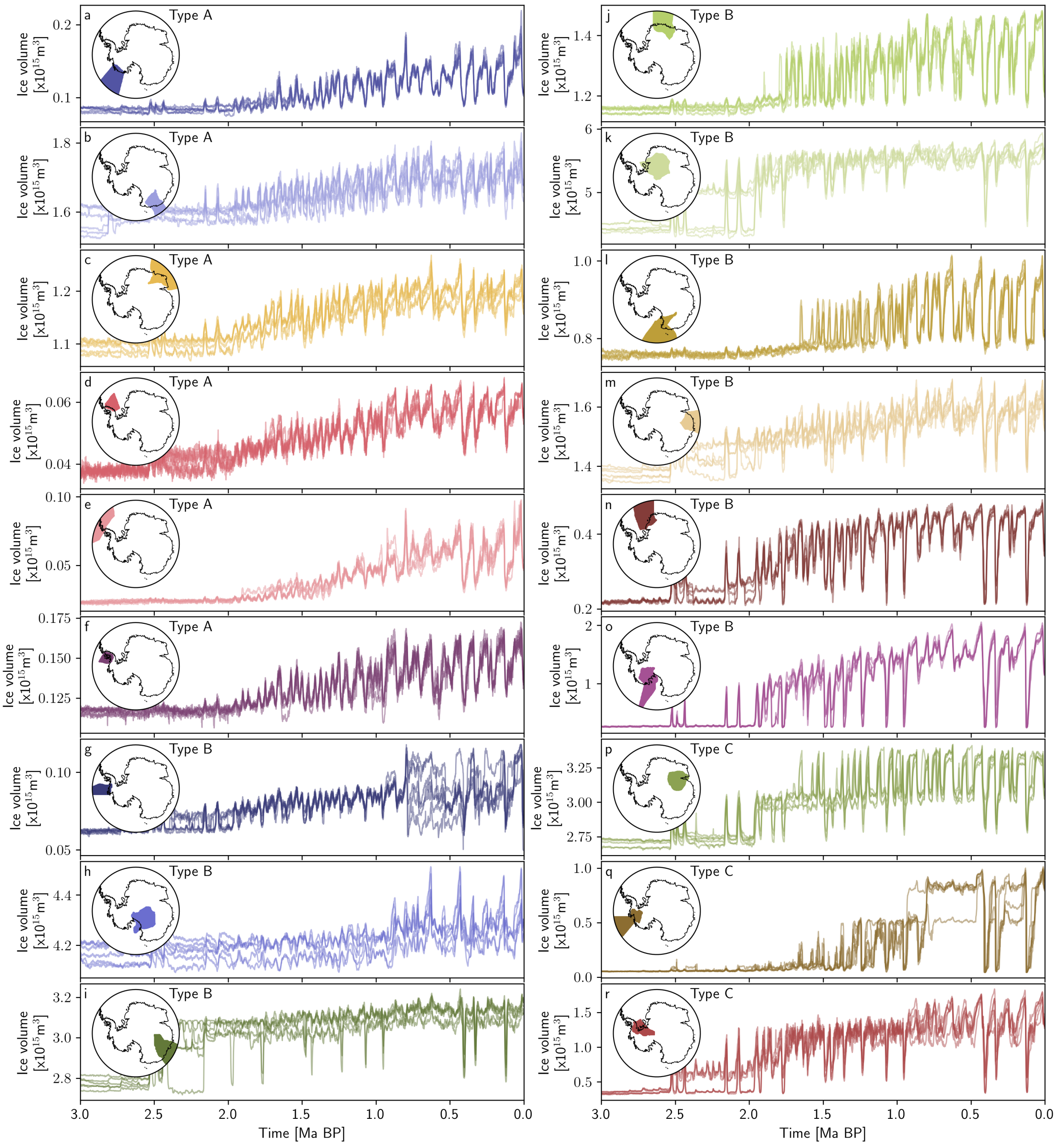

Figure S2: Grounded ice volume over the last 3 Myrs for all Antarctic drainage basins. a)-r) grounded ice volume for all six model simulations for each individual drainage basin over the last three million years. For each of the 18 drainage basins the associated basin type is stated together with an accompanying map highlighting the basins location.

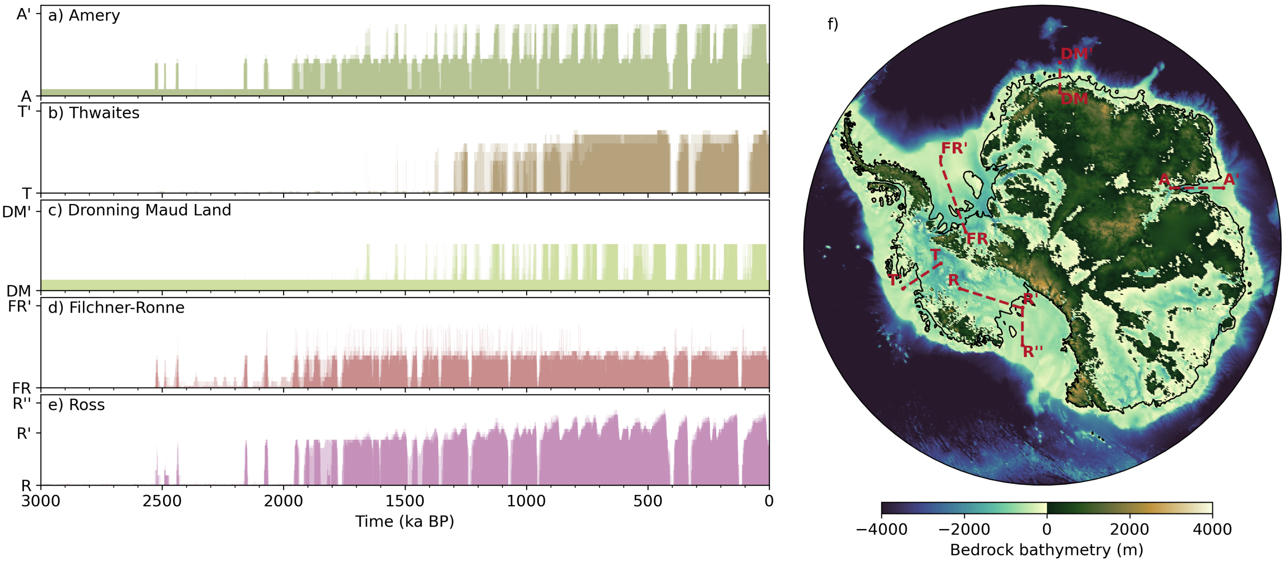

Figure S3: Grounding line evolution over the last 3 Myrs. Temporal evolution of the simulated grounding line position of all six simulations along transects of the (a) Amery, (b) Thwaites, (c) Dronning Maud, (d) Ronne and (e) Ross basin over the last 3 Myrs. (f) Bedrock bathymetry [1], transects (dashed red lines) in the four basins (e.g., A, A', etc.), and observed grounding line position (solid black line) around Antarctica.

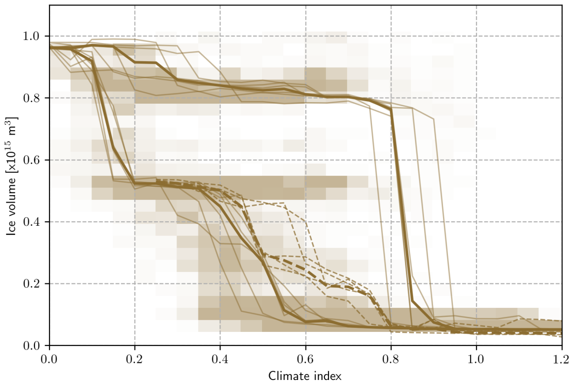

Figure S4: Hysteresis of the intermediate Thwaites state. Phase space density of the transient 3 million years ice sheet model simulation together with stepwise hysteresis of grounded ice volume for the Thwaites basin. Solid lines show hysteresis with reversal after Last Glacial Maximum climate conditions have been reached. Dashed lines show reversal from climate index of 0.25. Thick lines mark the median of all individual simulations (thin lines). The comparison between the transient simulation phase space density and the "equilibrium" hysteresis illustrates that the intermediate state is resilient towards millennial to orbital-scale overshooting.

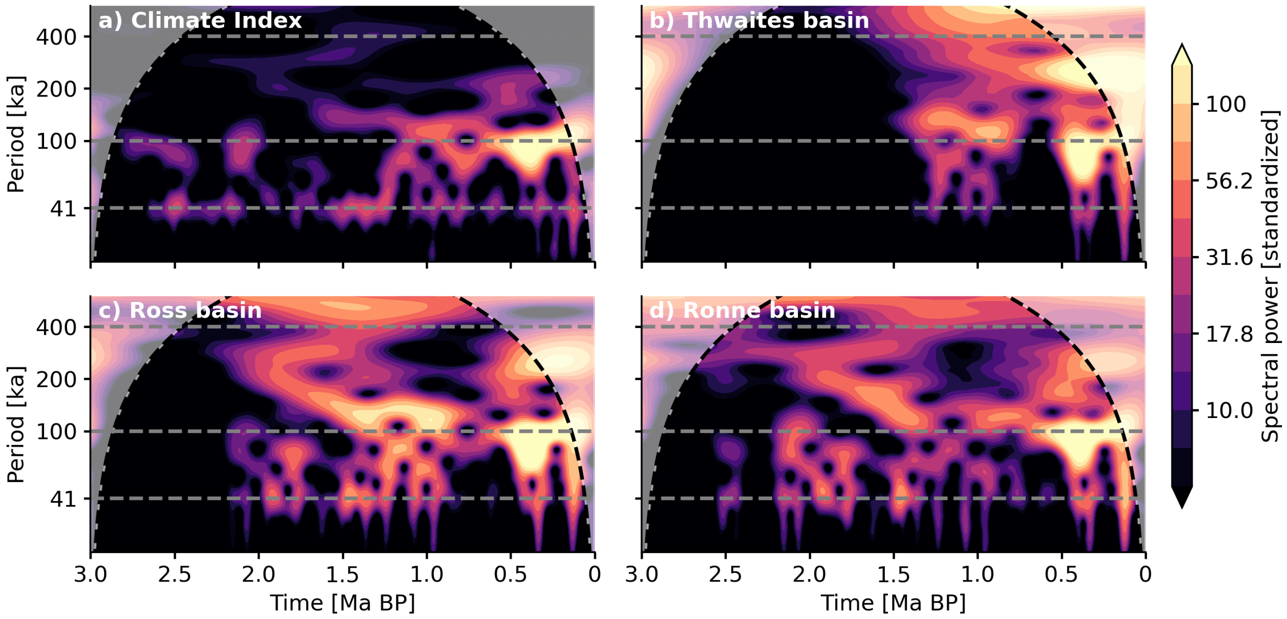

Figure S5: Evolutionary spectrum of the climate index and grounded ice volume. Mean evolutionary spectrum of the a) climate index and the grounded ice volume of all six simulations for b) Thwaites, c) Ross, and d) Ronne basins. Dashed gray lines indicate 41, 100 and 400 kyr periods. Shaded regions and black dashed lines outline the cone of influence.

2 Comparison to present-day observations

To validate our model setup, we compare ice thickness, surface velocities, and grounding line position at the end of our 3-million-year simulation with present-day observations (see Fig. S6). All simulations tend to accumulate more ice than currently observed around mountain ranges (e.g. Transantarctic mountains, Ellsworth mountains etc.) where ice flow is mainly driven by small fast outlet glaciers. The width of those outlet glaciers are often smaller or of similar size compared to our model resolution of 16 km, leading to a poorly resolved ice flow in these areas. We further find overall lower ice thickness in the central WAIS compared to present-day observations. This can be attributed to a poorly resolved basal hydrology as well as our choice of the till friction angle ϕ used to calculate the yield stress at the base. Here we linearly interpolate between ϕ_{min} and ϕ_{max} for bed elevations between b_{min} and b_{max} with the gradient $M = (\phi_{max} - \phi_{min})/(b_{max} - b_{min})$ by

$$\phi(x,y) = \begin{cases} \phi_{min}, & b(x,y) \leq b_{min}, \\ \phi_{min} + (b(x,y) - b_{min})M, & b_{min} < b(x,y) < b_{max}, \\ \phi_{max}, & b_{max} \leq b(x,y) \end{cases} \tag{1}$$

following Aschwanden et al. [2], Winkelmann et al. [3], Martin et al. [4], which might result in too little basal friction at the ice base in central WAIS. Inverting or iteratively optimizing for the basal friction [5, 6] might achieve better fits between simulated and observed ice sheet thickness. However, those methods might overcompensate for other errors that could result in ice thickness deviations [7] and therefore might not generalize well for past climate conditions. In summary, our simulations exhibit an ice thickness RMSE of 333 m which is still within the range of the Ice Sheet Model Intercomparison for CMIP6 (ISMIP6) [8]. In terms of simulated surface velocities, our simulations perform generally well and our calculated RMSE of 152 m/yr fits into the lower end of the IMSIP6 ensemble [8]. Yet, we observe substantially lower ice flow in the Filchner-Ronne and western Ross ice shelf, which might also be an explanation for increased ice thickness in surrounding areas.

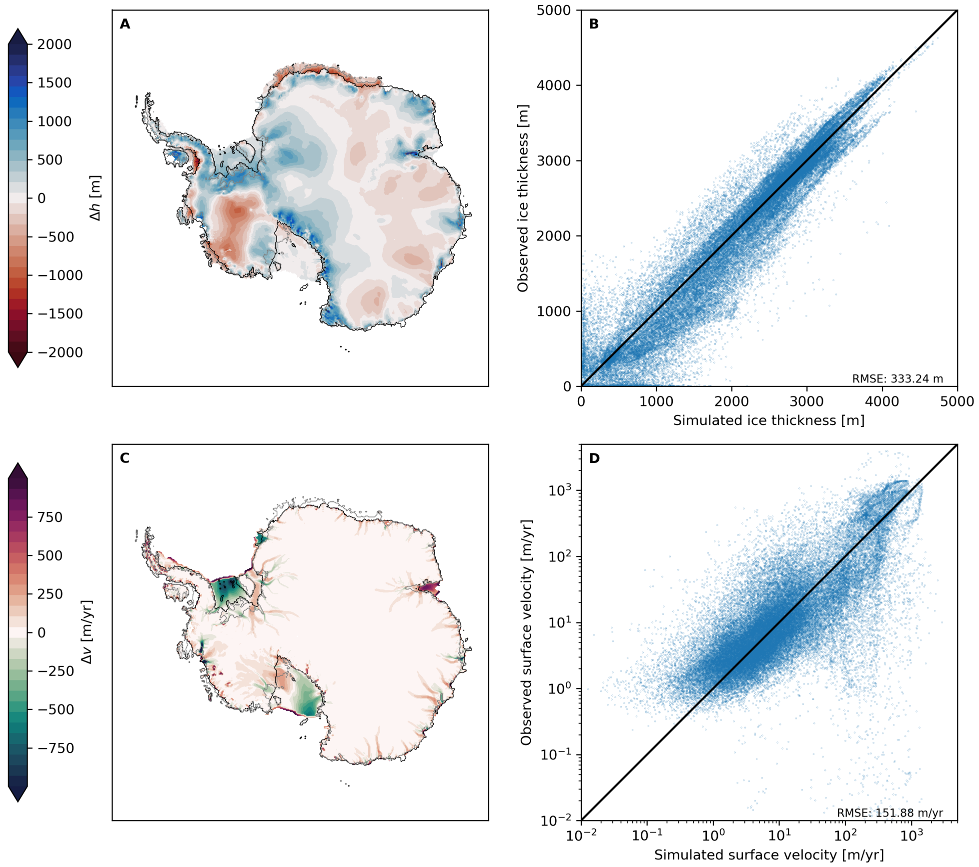

Figure S6: Evaluation of simulations vs. modern day observations. (A) Mean ice thickness difference between the end of the 3 Myrs simulation and present-day observations [1] regridded to 16 km resolution. (B) Scatter plot between simulated and observed ice sheet thickness. (C) Difference between simulated and observed surface velocities. (D) Scatter plot between simulated and observed [9] surface velocity. The black line is the identity line.

3 Past ice sheet state and proxy comparison

Figure S7 illustrates surface height anomalies relative to the end of simulation state, along with simulated grounding line positions for the Pliocene epoch (3 Ma BP), the LIG (120 ka BP), and the LGM (19 ka BP). While the WAIS exhibits substantial ice loss during both the Pliocene and LIG, the EAIS displays lower ice sheet height during the Pliocene compared to the LIG. This discrepancy may be attributed to generally higher temperatures, particularly in the ocean (see Fig. S11), leading to increased discharge. Additionally, prolonged exposure to substantially warmer than present-day climate conditions during the Pliocene (300 kyr spinup) allowed the ice sheet to approach its equilibrium state more closely than during the LIG. The LGM shows a higher and extended WAIS, accompanied with substantial grounding line advance in the Ross and Filchner-Ronne ice shelves. Similar patterns are also simulated for the costal margins of the EAIS. However, the central EAIS also exhibits a lower ice sheet height compared to end of simulation state, which is driven by the reduced precipitation and subsequently lower surface mass balance during the colder climate conditions in that region.

On a local scale, we illustrate simulated ice thickness as well as the flotation criterion close to the ANDRILL AND-1B [10] location (see Fig. S8). From 3.0 Ma to 1.7 Ma BP, open ocean conditions alternated with floating shelf ice in agreement with reconstructions from the AND-1B sediment core [11]. From around 1.7 Ma to 1.6 Ma BP we observe a regime shift towards predominately grounded ice, which is temporally interrupted by grounding line retreat and open ocean conditions during warm interglacials. In accordance with findings from McKay et al. [11] we find 7 periods of grounding line retreat after the MPT. Nevertheless, the complexity of the AND-1B sediment core [10, 11] and the complicated geography and ice flow pattern around Ross Island limit the comparison of our modeling results to reconstructions. To enable a direct comparison between ice sheet model simulations and reconstructions from AND-1B, a significantly higher model resolution, capable of resolving narrow outlet glaciers across the Transantarctic Mountains, would be required, exceeding computational capacities for a 3 Myr long simulation.

Further, we validate our simulated temperature profile against borehole temperatures at the Dome Fuji (CF), EPICA Dronning Maud Land (EDML) and EPICA Dome C (EDC) ice core locations (see. Fig. S9). Our simulations perform well in reproducing the borehole temperature profile below 1000 meters. Only for EDML a slight warm bias is present, which could result from a bias within the applied geothermal heat flux forcing. In the upper 1000 m we observe a warm bias in our simulations for all three ice core locations, which might originate from a combination of biases within the applied temperature forcing from both the regional (RACMO) and global (COSMOS) climate model and the modeled ice thickness.

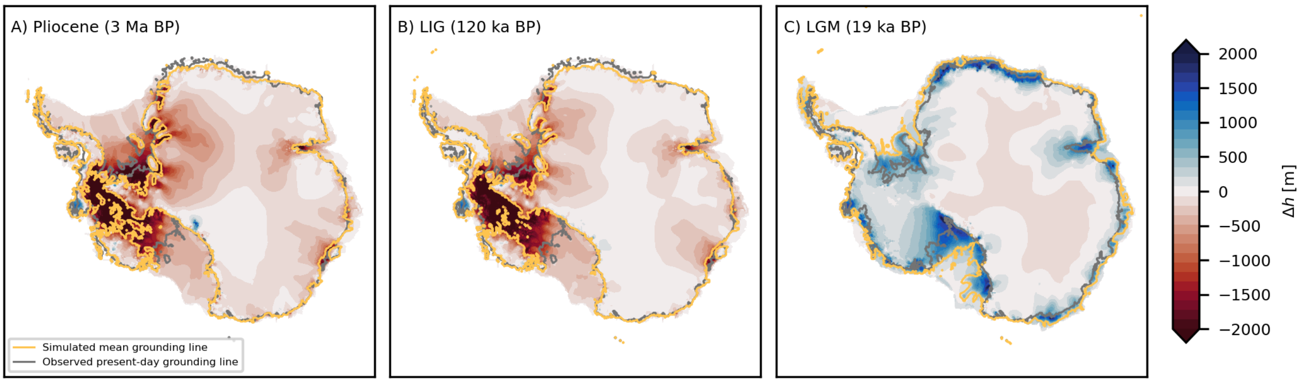

Figure S7: Thickness and grounding line differences to the present-day state. Mean ice thickness difference with respect to the end of simulation and observed [1] (gray) and simulated (yellow) present-day grounding line position for (A) Pliocene (3Ma BP), (B) Last Interglacial (LIG, 120 ka BP) and (C) Last Glacial Maximum (LGM, 19 ka BP). For the Pliocene and LIG warm climate conditions, increased melt of marine ice results in collapse of the West Antarctic Ice Sheet. Increased ice discharge also results in lower East Antarctic Ice Sheet height. For the LGM, the lower sea-levels and colder ocean temperatures permit a substantial grounding line advance into the Ross and Filchner-Ronne ice shelve. For east Antarctic ice margins an increase in ice thickness due to lower discharge and melt is observed. However, central East Antarctica shows smaller ice sheet thickness due to decreased precipitation rates as a consequence of the colder climate.

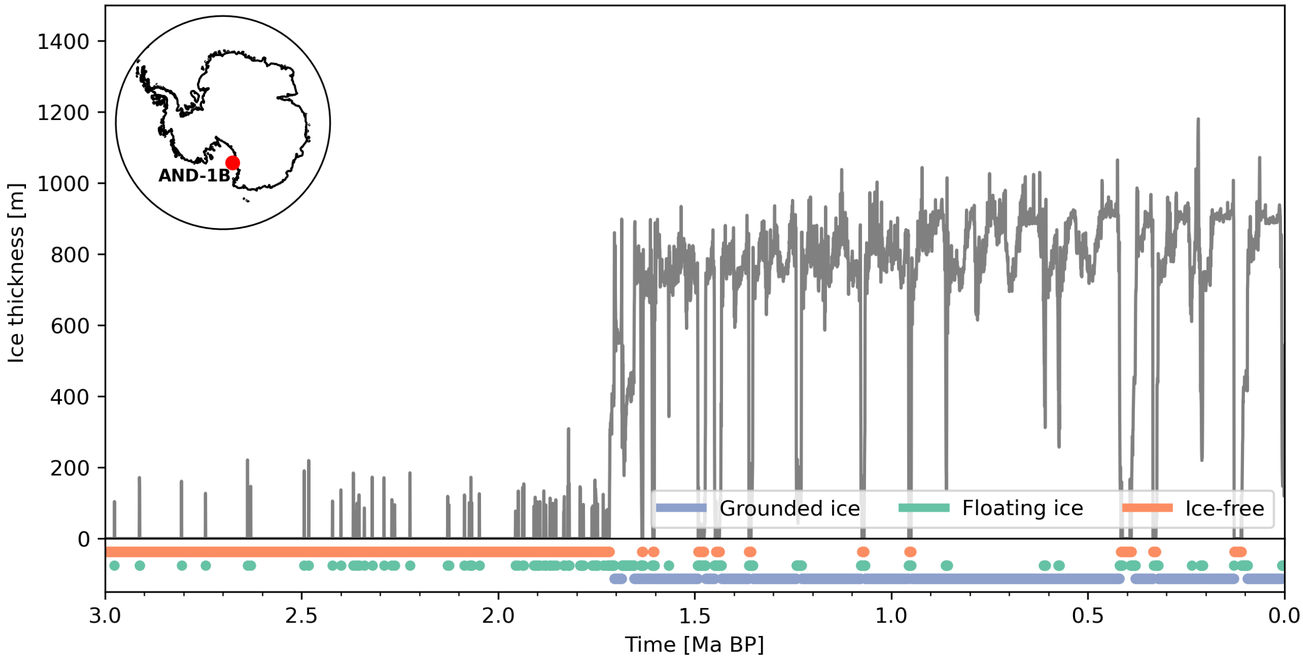

Figure S8: Ice sheet evolution at the AND-1B location. Simulated ice thickness and floating condition for *Config. 1* (see Tab. A1) retrieved from two grid boxes east of the AND-1B location to avoid poorly resolved Ross island shore line.

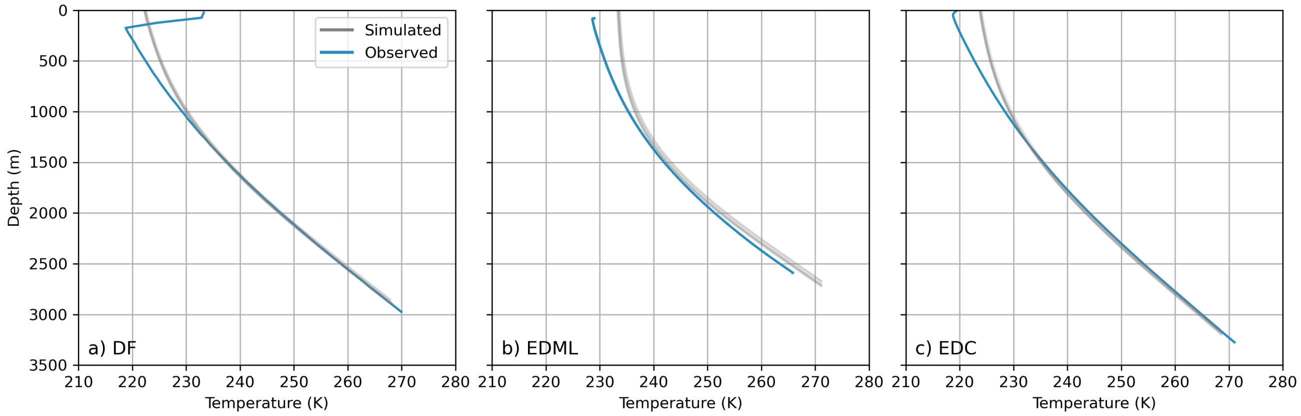

Figure S9: Simulated and observed [12] borehole temperature at selected east Antarctic ice core drilling sites. (a) Dome Fuji, (b) EPICA Dronning Maud Land, (c) EPICA Dome C.

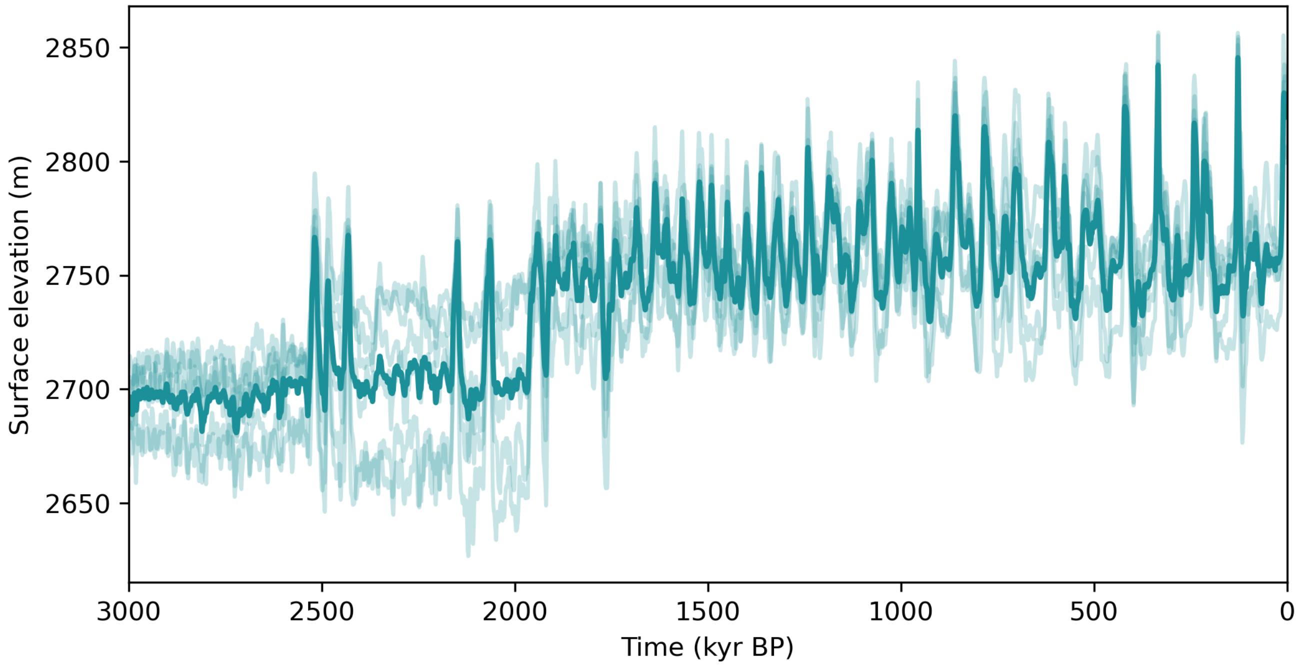

Figure S10: Surface elevation at the EDML ice core site throughout the last three million years. Thick line shows the ensemble mean, shaded lines individual ensemble members.

4 Climate forcing

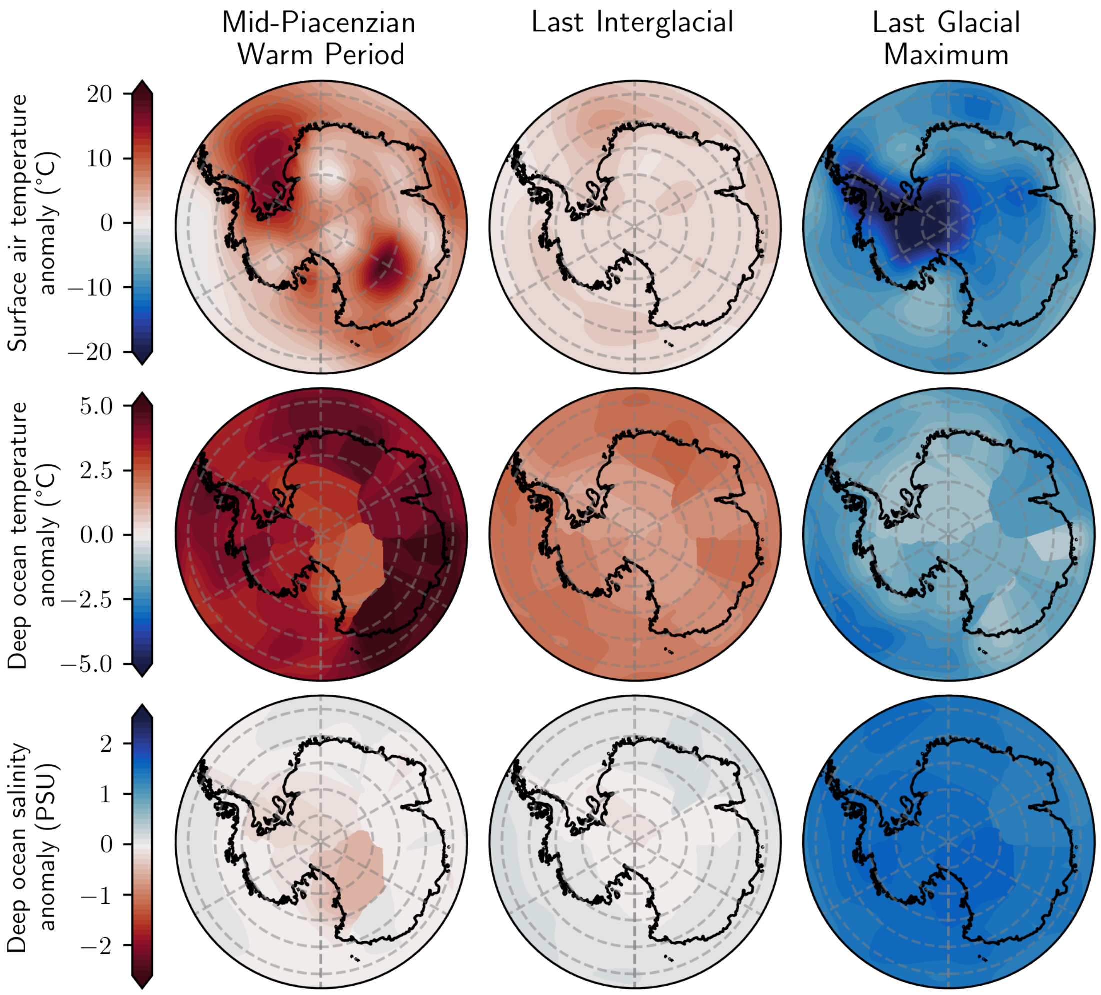

Figure S11: Utilized forcing snapshots. Snapshots of the applied near surface air temperature anomalies (top row), deep ocean (300-700 m) temperature (mid row) and salinity (bottom row) anomalies for the mid-Piacenzian Warm Period (mPWP, 3.3-3.0 Ma BP) [13] (Eoi400; left column), Last Interglacial [14] (LIG-ctl; mid column) and Last Glacial Maximum [15] (LGM-ctl; right column).

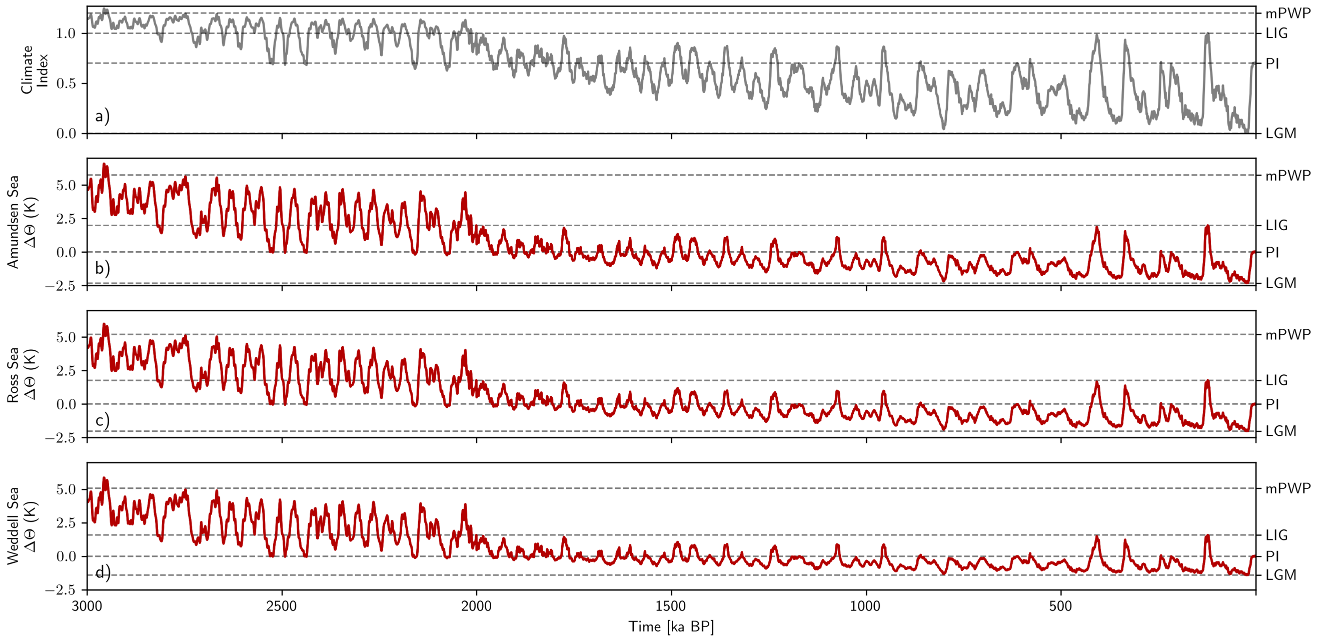

Figure S12: Oceanic temperature evolution. Temporal evolution of (a) climate index and mean deep ocean (300-700 m) temperature anomaly in (b) Amundsen, (c) Ross and (d) Weddell Seas. The right axis marks mean ocean temperatures for the mid-Piacenzian Warm Period (mPWP, 3.3-3.0 Ma BP), the Last Interglacial (LIG), the pre-industrial reference value (PI) and the Last Glacial Maximum (LGM). Please note that the increased variability before 2 Ma BP occurs due to the large difference of ocean temperature between the LIG and mPWP as shown in Fig. S11.

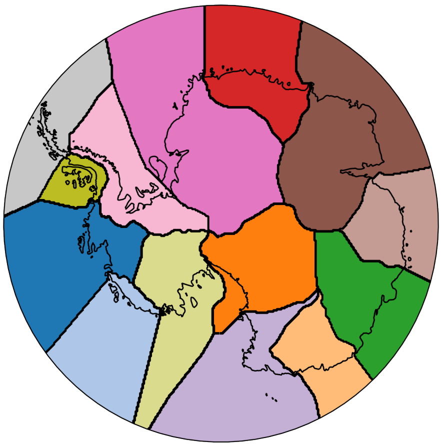

Figure S13: Modified IMBIE [9] basins used for extrapolating the ocean forcing as described in the Methods. In comparison to the IMBIE basins we have merged basins 14 & 15, 6 & 11, 8 & 13 and 1 & 9 (numbering as in [9]) to ensure that all basins are connected to the open ocean.

5 Sea-Level Forcing

Sea-level reconstructions were derived from the LR04 benthic δ^{18}O stack [16], which captures combined influences of ice volume and deep-ocean temperature. We applied a linear calibration following Foster and Rohling [17], expressed as:

$$SL = (3.23 - \delta^{18}O) \times \frac{6.5\text{ m}}{0.1} \tag{2}$$

This yields sea-level estimates of approximately $SL_{\mathrm{LGM}} = -115$ m at the Last Glacial Maximum and $SL_{\mathrm{LIG}} = +8$ m during the Last Interglacial, consistent with independent reconstructions [18, 17, 19]. The resulting sea level forcing is illustrated in Fig. S14.

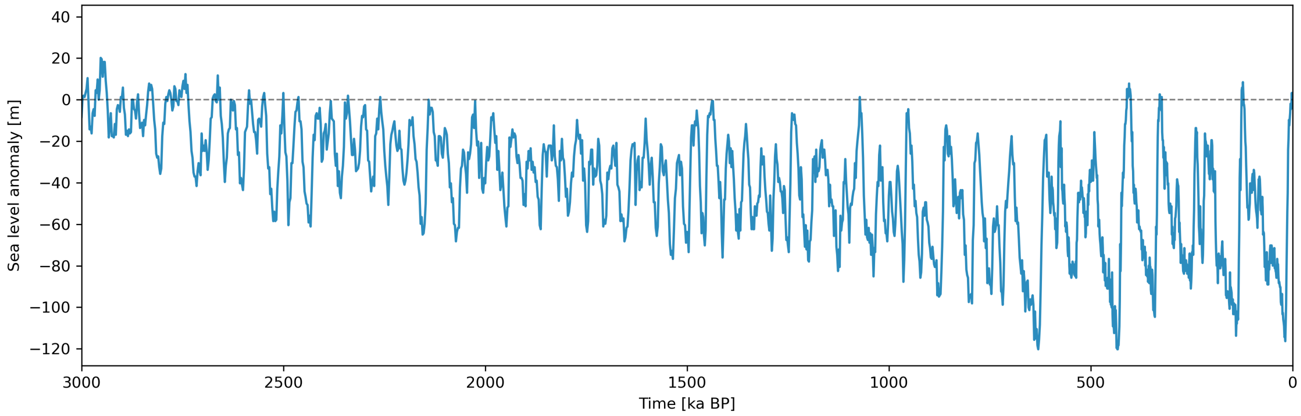

Figure S14: Sea-level forcing applied in the transient three-million-year simulations. Sea-level estimates were obtained from the benthic $\delta^{18}O$ record [16], using equation 2.

6 Stability of the Wilkes subglacial basin

In none of our simulations do we observe a large-scale disintegration of the Wilkes Subglacial Basin. As shown by Mengel and Levermann [20], the basin's stability is primarily controlled by the position of the grounding line at distinct ice plugs located at the Ninnis, Cook, and Mertz glaciers. Throughout our simulations, the George V coast grounding line remains slightly advanced under the warm climatic conditions of the Pliocene and the LIG (see Fig. S15), resulting in a persistently stable Wilkes Basin across all simulations.

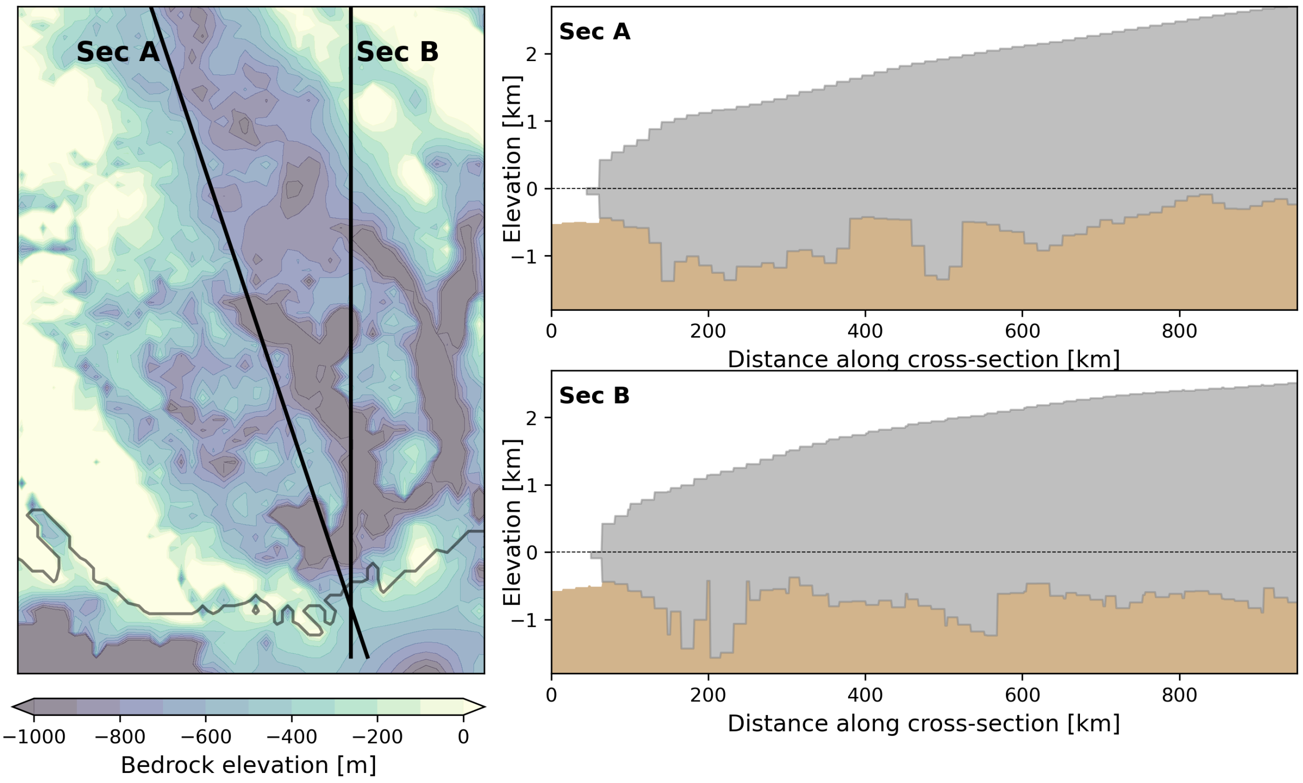

Figure S15: Ice sheet cross-section across Wilkes basin. Bedrock elevation and cross-sections through Wilkes subglacial basin and through the ice plug as in [20] during the Last Interglacial (120 ka BP) simulated under model configuration 1. The ice sheet configuration in both cross-sections reveals that the grounding line has not reached an unstable configuration.

7 On the origin of the thresholds in the Thwaites and Pine Island basin

To assess the origin of the unique thresholds within the Thwaites and Pine Island glacier we have illustrated the ice-bedrock configurations along two cross-sections through the respective glaciers. Both the simulated present-day configuration and the LGM and Proto-WAIS / intermediate states are shown in Fig. S16. As becomes apparent, in all configurations the ice sheet seems to rest on a prograde slope, resulting in a stable grounding line position. Especially, the intermediate state of Thwaites glacier is in a partly advanced configuration (wrt. the the onset the prograde slope) on this prograde slope, which might explain its enhanced stability under paleo-overshooting on glacial-interglacial time scales before and during the MPT. The intermediate / Proto-WAIS state shows a similar but more complicated picture for Pine Island (Sec B). While the grounding line along the cross-section is also situated on a prograde bedrock slope, the grounding line around the Pine Island subglacial trough likewise appears to rest on a prograde slope (see bedrock bathymetry), which likely constitutes the substantial threshold controlling the secondary advance of the grounding line.

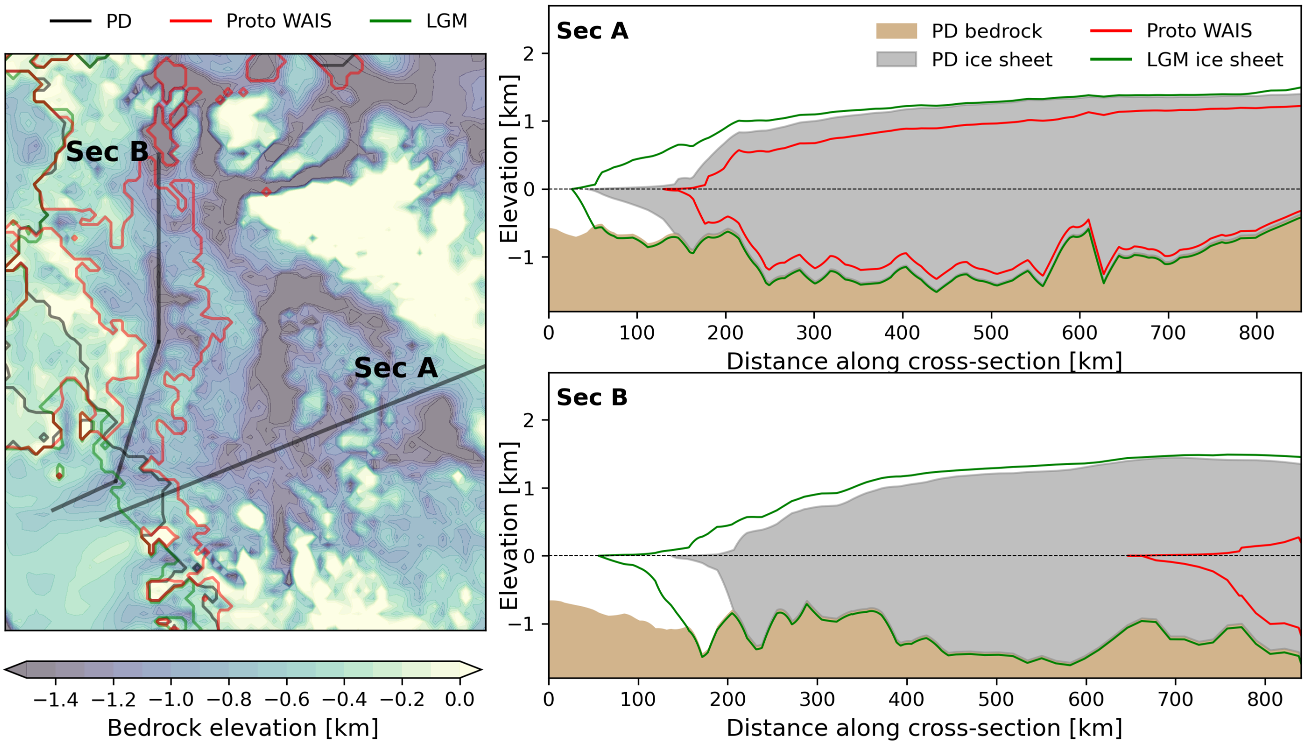

Figure S16: Ice sheet cross-section across Thwaites and Pine Island glacier. Bedrock elevation and cross-sections through Thwaites (Sec A) and Pine Island (Sec B) subglacial basin for simulated present-day (PD) ice sheet (grey) and bedrock (brown) configurations, as well as the ice sheet configuration during the Last Glacial Maximum (LGM, green) and the Proto West Antarctic Ice Sheet (WAIS) state (red) simulated under model configuration 1. In all cases the grounding line stabilizes at the onset of a prograde bedrock slope that limits the advancement of the grounding line. The grounding line in the Proto WAIS configuration (Sec A) seems to be also positioned on a prograde bedrock slope that might support the increased stability during past "overshooting" events of that specific basin.

8 Potential mechanisms for enhanced long term variability

The enhancement of 100 kyr variability within the Thwaites basin seems to originate from a combination of the unique climatic thresholds and the "overshooting stability" of the intermediate / proto-WAIS state (see Fig. 4). Under the "right" climatic conditions this can lead to skipping of interglacial collapses, enhancing variability at lower frequencies. As the climate index time series used in this study contains variability at various frequencies, we have performed an additional simulation with a synthetic climate index using the long-term trend of the climate index time series with an artificial 41 kyr sinusoidal signal. We added the synthetic climate index and the resulting ice volume evolution in the Thwaites basin for model configuration No. 1 to supplementary Fig. S17. We infer that for the synthetic climate index, oscillating between interglacial values of 0.60-0.67 and glacial values of 0.20-0.27, skipping of interglacial ice sheet collapse can be observed, although the occurrence of "skipping" does not seem to be of deterministic nature. We attribute the latter to the complexity of the three-dimensional ice flow within the basin.

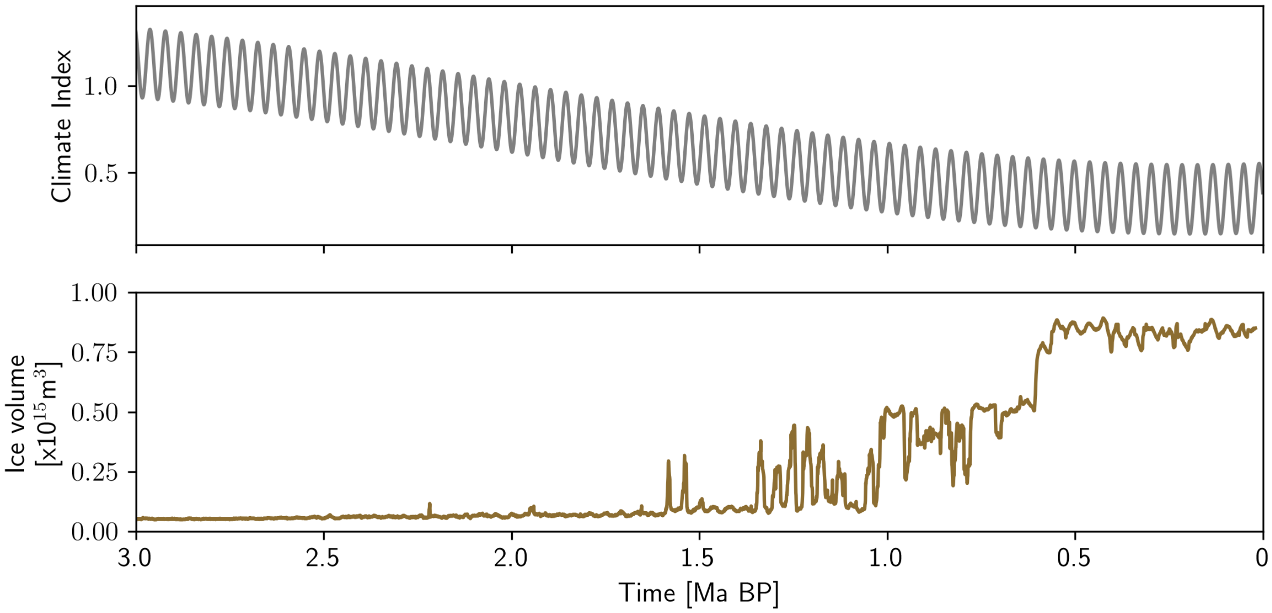

Figure S17: Tracing enhancement of 100 kyr variability within the Thwaites basin. We show a synthetic climate Index created by adding a 41 kyr sinusoidal onto the long-term trend of the original climate index (top) and the resulting ice volume within the Thwaites basin (bottom). For climate index values between 0.20-0.27 (glacial) and 0.60-0.67 (interglacial), the unique thresholds in the basin and their temporal stability can lead to skipping of interglacial collapses, ultimately leading to enhanced lower-frequency (100 kyr) variability.

References

[1] Mathieu Morlighem, Eric Rignot, Tobias Binder, Donald Blankenship, Reinhard Drews, Graeme Eagles, Olaf Eisen, Fausto Ferraccioli, René Forsberg, Peter Fretwell, Vikram Goel, Jamin S. Greenbaum, Hilmar Gudmundsson, Jingxue Guo, Veit Helm, Coen Hofstede, Ian Howat, Angelika Humbert, Wilfried Jokat, Nanna B. Karlsson, Won Sang Lee, Kenichi Matsuoka, Romain Millan, Jeremie Mouginot, John Paden, Frank Pattyn, Jason Roberts, Sebastian Rosier, Antonia Ruppel, Helene Seroussi, Emma C. Smith, Daniel Steinhage, Bo Sun, Michiel R. van den Broeke, Tas D. van Ommen, Melchior van Wessem, and Duncan A. Young. Deep glacial troughs and stabilizing ridges unveiled beneath the margins of the Antarctic ice sheet. *Nature Geoscience*, 13(2):132–137, February 2020. ISSN 1752-0908. doi: 10.1038/s41561-019-0510-8. URL `https://www.nature.com/articles/s41561-019-0510-8`. Number: 2 Publisher: Nature Publishing Group.

[2] A. Aschwanden, G. Aalgeirsdóttir, and C. Khroulev. Hindcasting to measure ice sheet model sensitivity to initial

states. *The Cryosphere*, 7(4):1083–1093, July 2013. ISSN 1994-0416. doi: 10.5194/tc-7-1083-2013. URL https://tc.copernicus.org/articles/7/1083/2013/. Publisher: Copernicus GmbH.

[3] R. Winkelmann, M. A. Martin, M. Haseloff, T. Albrecht, E. Bueler, C. Khroulev, and A. Levermann. The Potsdam Parallel Ice Sheet Model (PISM-PIK) – Part 1: Model description. *The Cryosphere*, 5(3):715–726, September 2011. ISSN 1994-0416. doi: 10.5194/tc-5-715-2011. URL https://tc.copernicus.org/articles/5/715/2011/tc-5-715-2011.html. Publisher: Copernicus GmbH.

[4] M. A. Martin, R. Winkelmann, M. Haseloff, T. Albrecht, E. Bueler, C. Khroulev, and A. Levermann. The Potsdam Parallel Ice Sheet Model (PISM-PIK) – Part 2: Dynamic equilibrium simulation of the Antarctic ice sheet. *The Cryosphere*, 5(3):727–740, September 2011. ISSN 1994-0424. doi: 10.5194/tc-5-727-2011. URL https://tc.copernicus.org/articles/5/727/2011/.

[5] D. Pollard and R. M. DeConto. A simple inverse method for the distribution of basal sliding coefficients under ice sheets, applied to Antarctica. *The Cryosphere*, 6(5):953–971, September 2012. ISSN 1994-0424. doi: 10.5194/tc-6-953-2012. URL https://tc.copernicus.org/articles/6/953/2012/.

[6] Dawei Li, Robert M. DeConto, and David Pollard. Climate model differences contribute deep uncertainty in future Antarctic ice loss. *Science Advances*, 9(7):eadd7082, February 2023. doi: 10.1126/sciadv.add7082. URL https://www.science.org/doi/10.1126/sciadv.add7082. Publisher: American Association for the Advancement of Science.

[7] Christian Wirths, Thomas F. Stocker, and Johannes C. R. Sutter. The influence of present-day regional surface mass balance uncertainties on the future evolution of the Antarctic Ice Sheet. *The Cryosphere*, 18(9):4435–4462, September 2024. ISSN 1994-0416. doi: 10.5194/tc-18-4435-2024. URL https://tc.copernicus.org/articles/18/4435/2024/. Publisher: Copernicus GmbH.

[8] Hélène Seroussi, Sophie Nowicki, Antony J. Payne, Heiko Goelzer, William H. Lipscomb, Ayako Abe-Ouchi, Cécile Agosta, Torsten Albrecht, Xylar Asay-Davis, Alice Barthel, Reinhard Calov, Richard Cullather, Christophe Dumas, Benjamin K. Galton-Fenzi, Rupert Gladstone, Nicholas R. Golledge, Jonathan M. Gregory, Ralf Greve, Tore Hattermann, Matthew J. Hoffman, Angelika Humbert, Philippe Huybrechts, Nicolas C. Jourdain, Thomas Kleiner, Eric Larour, Gunter R. Leguy, Daniel P. Lowry, Chistopher M. Little, Mathieu Morlighem, Frank Pattyn, Tyler Pelle, Stephen F. Price, Aurélien Quiquet, Ronja Reese, Nicole-Jeanne Schlegel, Andrew Shepherd, Erika Simon, Robin S. Smith, Fiammetta Straneo, Sainan Sun, Luke D. Trusel, Jonas Van Breedam, Roderik S. W. van de Wal, Ricarda Winkelmann, Chen Zhao, Tong Zhang, and Thomas Zwinger. ISMIP6 Antarctica: a multi-model ensemble of the Antarctic ice sheet evolution over the 21st century. *The Cryosphere*, 14(9):3033–3070, September 2020. ISSN 1994-0416. doi: 10.5194/tc-14-3033-2020. URL https://tc.copernicus.org/articles/14/3033/2020/. Publisher: Copernicus GmbH.

[9] E. Rignot, J. Mouginot, and B. Scheuchl. Ice Flow of the Antarctic Ice Sheet. *Science*, 333(6048):1427–1430, September 2011. doi: 10.1126/science.1208336. URL https://www.science.org/doi/10.1126/science.1208336. Publisher: American Association for the Advancement of Science.

[10] T. Naish, R. Powell, R. Levy, G. Wilson, R. Scherer, F. Talarico, L. Krissek, F. Niessen, M. Pompilio, T. Wilson, L. Carter, R. DeConto, P. Huybers, R. McKay, D. Pollard, J. Ross, D. Winter, P. Barrett, G. Browne, R. Cody, E. Cowan, J. Crampton, G. Dunbar, N. Dunbar, F. Florindo, C. Gebhardt, I. Graham, M. Hannah, D. Hansaraj, D. Harwood, D. Helling, S. Henrys, L. Hinnov, G. Kuhn, P. Kyle, A. Läufer, P. Maffioli, D. Magens, K. Mandernack, W. McIntosh, C. Millan, R. Morin, C. Ohneiser, T. Paulsen, D. Persico, I. Raine, J. Reed, C. Riesselman, L. Sagnotti, D. Schmitt, C. Sjunneskog, P. Strong, M. Taviani, S. Vogel, T. Wilch, and T. Williams. Obliquity-paced Pliocene West Antarctic ice sheet oscillations. *Nature*, 458(7236):322–328, March 2009. ISSN 1476-4687. doi: 10.1038/nature07867. URL https://www.nature.com/articles/nature07867. Publisher: Nature Publishing Group.

[11] Robert McKay, Tim Naish, Ross Powell, Peter Barrett, Reed Scherer, Franco Talarico, Philip Kyle, Donata Monien, Gerhard Kuhn, Chris Jackolski, and Trevor Williams. Pleistocene variability of Antarctic Ice Sheet extent in the Ross Embayment. *Quaternary Science Reviews*, 34:93–112, February 2012. ISSN 0277-3791. doi: 10.1016/j.quascirev.2011.12.012. URL https://www.sciencedirect.com/science/article/pii/S0277379111004057.

[12] Benoit S. Lecavalier, Lev Tarasov, Greg Balco, Perry Spector, Claus-Dieter Hillenbrand, Christo Buizert, Catherine Ritz, Marion Leduc-Leballeur, Robert Mulvaney, Pippa L. Whitehouse, Michael J. Bentley, and Jonathan Bamber. Antarctic Ice Sheet paleo-constraint database. *Earth System Science Data*, 15(8):3573–3596, August 2023. ISSN

1866-3508. doi: 10.5194/essd-15-3573-2023. URL https://essd.copernicus.org/articles/15/3573/2023/. Publisher: Copernicus GmbH.

[13] Christian Stepanek, Eric Samakinwa, Gregor Knorr, and Gerrit Lohmann. Contribution of the coupled atmosphere–ocean–sea ice–vegetation model COSMOS to the PlioMIP2. *Climate of the Past*, 16(6):2275–2323, November 2020. ISSN 1814-9324. doi: 10.5194/cp-16-2275-2020. URL https://cp.copernicus.org/articles/16/2275/2020/. Publisher: Copernicus GmbH.

[14] Madlene Pfeiffer and Gerrit Lohmann. Greenland Ice Sheet influence on Last Interglacial climate: global sensitivity studies performed with an atmosphere–ocean general circulation model. *Climate of the Past*, 12(6):1313–1338, June 2016. ISSN 1814-9324. doi: 10.5194/cp-12-1313-2016. URL https://cp.copernicus.org/articles/12/1313/2016/. Publisher: Copernicus GmbH.

[15] X. Zhang, G. Lohmann, G. Knorr, and X. Xu. Different ocean states and transient characteristics in Last Glacial Maximum simulations and implications for deglaciation. *Climate of the Past*, 9(5):2319–2333, October 2013. ISSN 1814-9324. doi: 10.5194/cp-9-2319-2013. URL https://cp.copernicus.org/articles/9/2319/2013/. Publisher: Copernicus GmbH.

[16] Lorraine E. Lisiecki and Maureen E. Raymo. A Pliocene-Pleistocene stack of 57 globally distributed benthic 18O records. *Paleoceanography*, 20(1), 2005. ISSN 1944-9186. doi: 10.1029/2004PA001071. URL https://onlinelibrary.wiley.com/doi/abs/10.1029/2004PA001071. _eprint: https://onlinelibrary.wiley.com/doi/pdf/10.1029/2004PA001071.

[17] Gavin L. Foster and Eelco J. Rohling. Relationship between sea level and climate forcing by CO_2 on geological timescales. *Proceedings of the National Academy of Sciences*, 110(4):1209–1214, January 2013. doi: 10.1073/pnas.1216073110. URL https://www.pnas.org/doi/abs/10.1073/pnas.1216073110. Company: National Academy of Sciences Distributor: National Academy of Sciences Institution: National Academy of Sciences Label: National Academy of Sciences Publisher: Proceedings of the National Academy of Sciences.

[18] E. J. Rohling, K. Grant, M. Bolshaw, A. P. Roberts, M. Siddall, Ch Hemleben, and M. Kucera. Antarctic temperature and global sea level closely coupled over the past five glacial cycles. *Nature Geoscience*, 2(7):500–504, July 2009. ISSN 1752-0908. doi: 10.1038/ngeo557. URL https://www.nature.com/articles/ngeo557. Publisher: Nature Publishing Group.

[19] A. Dutton, A. E. Carlson, A. J. Long, G. A. Milne, P. U. Clark, R. DeConto, B. P. Horton, S. Rahmstorf, and M. E. Raymo. Sea-level rise due to polar ice-sheet mass loss during past warm periods. *Science*, 349(6244):aaa4019, July 2015. doi: 10.1126/science.aaa4019. URL https://www.science.org/doi/10.1126/science.aaa4019. Publisher: American Association for the Advancement of Science.

[20] M. Mengel and A. Levermann. Ice plug prevents irreversible discharge from East Antarctica. *Nature Climate Change*, 4(6):451–455, June 2014. ISSN 1758-6798. doi: 10.1038/nclimate2226. URL https://www.nature.com/articles/nclimate2226. Publisher: Nature Publishing Group.
